# A quantitative study of neuronal nitric oxide synthase expression in laminae I–III of the rat spinal dorsal horn

**DOI:** 10.1016/j.neuroscience.2011.07.011

**Published:** 2011-09-29

**Authors:** T.C.P. Sardella, E. Polgár, M. Watanabe, A.J. Todd

**Affiliations:** a Institute of Neuroscience and Psychology, West Medical Building, University Avenue, University of Glasgow, Glasgow, G12 8QQ, UK; b Department of Anatomy, Hokkaido University School of Medicine, Sapporo 060-8638, Japan

**Keywords:** nNOS, GABA, PKCγ, inhibitory interneurons, confocal microscopy, cGMP, cyclic guanosine monophosphate, GAD67, 67 kDa molecular weight isoform of glutamic acid decarboxylase, GFP, green fluorescent protein, NADPH, reduced nicotinamide adenine dinucleotide phosphate, NK1r, neurokinin 1 receptor, nNOS, neuronal nitric oxide synthase, NO, nitric oxide, NPY, neuropeptide Y, PKCγ, protein kinase Cγ, sGC, soluble guanylate cyclase, VGAT, vesicular GABA transporter, VGLUT2, vesicular glutamate transporter 2

## Abstract

Nitric oxide produced by neuronal nitric oxide synthase (nNOS) in the spinal cord is required for development of hyperalgesia in inflammatory and neuropathic pain states. nNOS is expressed by some dorsal horn neurons, and an early study that used a histochemical method to identify these cells suggested that they were mainly inhibitory interneurons. We have carried out a quantitative analysis of nNOS-immunoreactivity in laminae I–III of the rat dorsal horn, to determine the proportion of inhibitory and excitatory neurons and axonal boutons that express the protein. nNOS was present in ∼5% of neurons in laminae I and III, and 18% of those in lamina II. Although most cells with strong nNOS immunostaining were GABA-immunoreactive, two-thirds of the nNOS-positive cells in lamina II and half of those in lamina III were not GABAergic, and some of these expressed protein kinase Cγ (PKCγ). We estimate that nNOS is present in 17–19% of the inhibitory interneurons in laminae I–II, and 6% of those in lamina III. However, our results suggest that nNOS is also expressed at a relatively low level by a significant proportion (∼17%) of excitatory interneurons in lamina II. nNOS was seldom seen in boutons that contained vesicular glutamate transporter 2, which is expressed by excitatory interneurons, but was co-localised with the vesicular GABA transporter (VGAT, a marker for GABAergic and glycinergic axons). nNOS was detected in 13% of VGAT boutons in lamina I and in 7–8% of those in laminae II–III. However, it was only found in 2–4% of the VGAT boutons that were presynaptic to PKCγ-expressing interneurons in this region. These results indicate that nNOS is more widely expressed than previously thought, being present in both inhibitory and excitatory neurons. They provide further evidence that axons of neurochemically defined populations of inhibitory interneuron are selective in their post-synaptic targets.

## Highlights

▶nNOS is expressed by nearly 20% of neurons in lamina II and 5% of those in laminae I and III. ▶The majority of nNOS neurons in laminae II and III are not GABAergic. ▶Between 7 and 13% of GABAergic boutons in laminae I–III are nNOS-immunoreactive. ▶nNOS axons form few synapses with PKCγ^+^ excitatory interneurons.

The spinal dorsal horn receives sensory inputs that are organised in a modality-specific way, with nociceptive and thermoreceptive afferents projecting mainly to the superficial laminae (I–II), and low-threshold mechanoreceptive afferents arborising in a region that extends from the inner half of lamina II (lamina IIi) to lamina VI (Light and Perl, 1979; Brown et al., 1981; Sugiura et al., 1986). Laminae I–III contain numerous densely packed neurons, and although some of those in laminae I and III project to the brain, the great majority of neurons in each of these laminae have axons that remain in the spinal cord, and are therefore classified as interneurons (Todd, 2010). Interneurons in laminae I–III are thought to modulate transmission of sensory information to the brain and to local reflex circuits.

Between 30 and 40% of the neurons in laminae I–III are inhibitory interneurons (Todd and Sullivan, 1990; Polgár et al., 2003). Virtually all of these are GABA-immunoreactive and many also contain high levels of glycine, which suggests that some inhibitory interneurons in this region use GABA and glycine as co-transmitters. Blocking GABAergic or glycinergic transmission at the spinal level leads to tactile allodynia (Yaksh, 1989; Sivilotti and Woolf, 1994), and inhibitory interneurons are thought to have several specific anti-nociceptive roles (Sandkühler, 2009). In addition, some of these cells are involved in prevention of itch (Ross et al., 2010). It is likely that distinct populations of inhibitory interneurons are responsible for these various functions. However, because of their morphological and electrophysiological diversity (Grudt and Perl, 2002; Heinke et al., 2004; Graham et al., 2007; Maxwell et al., 2007; Yasaka et al., 2007, 2010; Todd, 2010), it has proved difficult to identify discrete populations among these cells. An alternative approach to classification is based on expression of neurochemical markers, including neuropeptides and various proteins. For example, neuropeptide Y (NPY), galanin, parvalbumin and the neuronal form of nitric oxide synthase (nNOS) are expressed by different populations of GABAergic cells in laminae I–III (Antal et al., 1991; Rowan et al., 1993; Laing et al., 1994; Tiong et al., 2011).

Nitric oxide (NO) produced by nNOS plays an essential role in inflammatory and neuropathic pain states (Schmidtko et al., 2009), and it is therefore important to determine which neurons express nNOS. These were initially identified with a histochemical reaction for reduced nicotinamide adenine dinucleotide phosphate (NADPH) diaphorase (Valtschanoff et al., 1992a; Spike et al., 1993; Laing et al., 1994), and subsequently with nNOS antibodies (Dun et al., 1992, 1993; Valtschanoff et al., 1992b; Zhang et al., 1993; Herdegen et al., 1994; Saito et al., 1994; Wetts and Vaughn, 1994; Goff et al., 1998; Ruscheweyh et al., 2006). Spike et al. (1993) reported that the great majority of NADPH diaphorase-positive cells in laminae I–III (∼90%) were GABA-immunoreactive, and proposed that nNOS expression was largely restricted to inhibitory interneurons. This was consistent with the results of Valtschanoff et al. (1992b), who had found that all nNOS-immunoreactive axonal boutons in this region were enriched with GABA, but not glutamate.

There are still significant gaps in our knowledge about nNOS-expressing neurons in the dorsal horn. For example, there is little quantitative information about the proportion of cells or axonal boutons that contain nNOS, and this is needed in order to determine their contribution to neuronal circuits in the dorsal horn. In addition, Hughes et al. (2008) detected nNOS in some of the neurons in laminae Iii–III that expressed protein kinase Cγ (PKCγ), which is found in a sub-set of non-GABAergic neurons in this region (Polgár et al., 1999a), and this suggests that nNOS may be present in a significant number of excitatory interneurons. The aims of this study were to establish the proportion of neurons in laminae I–III that express nNOS and to test the hypothesis that nNOS is largely restricted to inhibitory interneurons in this region. We also determined the percentage of GABAergic boutons in each lamina that contained nNOS and tested whether PKCγ-expressing interneurons, which have been shown to be innervated by inhibitory interneurons that contain NPY (Polgár et al., 2011), were also targeted by nNOS-immunoreactive axons.

## Experimental procedures

All experiments were approved by the Ethical Review Process Applications Panel of the University of Glasgow, and were performed in accordance with the European Community directive 86/609/EC and the UK Animals (Scientific Procedures) Act 1986.

Spinal cords were obtained from 12 adult male Wistar rats (230–340 g; Harlan, Loughborough, UK). The animals were deeply anaesthetised with pentobarbitone (300 mg, i.p.; Rhone Merieux Ltd, Harlow, UK) and perfused through the left cardiac ventricle with fixative consisting of 4% freshly depolymerised formaldehyde in phosphate buffer. In three cases, the fixative also contained 0.2% glutaraldehyde. The fourth lumbar (L4) segment of the spinal cord was removed and stored at 4°C for 5–24 h in the same fixative, before being rinsed in phosphate buffer and cut into 60 μm thick transverse or sagittal sections with a vibratome. The sections were immersed for 30 min in 50% ethanol to enhance antibody penetration, and those that had been treated with glutaraldehyde-containing fixative were incubated for 30 min in 1% sodium borohydride. Transverse sections were used for all parts of the study, except the analyses of nNOS in GABAergic boutons presynaptic to PKCγ cells, and of the association between gephyrin puncta and GABAergic boutons (see below).

All immunocytochemical reactions were carried out at 4°C and antibodies were diluted in phosphate buffered saline that contained 0.3 M NaCl, without the addition of blocking serum. Unless otherwise stated, incubations in primary and secondary antibodies were for 3 days and overnight, respectively. Species-specific secondary antibodies (anti-IgG) were raised in donkey and conjugated to Alexa 488 (Invitrogen, Paisley, UK) or to Rhodamine Red or DyLight 649 (Jackson ImmunoResearch, West Grove, PA, USA). These were used at 1:500 (Alexa 488 and DyLight 649 conjugates) or 1:100 (Rhodamine Red conjugates).

### Proportion of neurons in laminae I–III that were nNOS-immunoreactive

Sections from three rats that had been fixed with formaldehyde were incubated in sheep anti-nNOS (Herbison et al., 1996) (1:2000) and monoclonal antibody NeuN (Millipore, Watford, UK, catalogue number MAB377; 1:500), which were revealed with secondary antibodies conjugated to Alexa 488 and DyLight 649, respectively. The sections were then incubated for 30 min at 37°C in Propidium Iodide (Sigma-Aldrich, Poole, UK; 1%) in the presence of RNase (Sigma-Aldrich; 10 mg/ml) to reveal cell nuclei (Todd et al., 1998; Polgár et al., 2011).

Two sections from each animal were selected before the nNOS immunostaining was viewed, and these were scanned with a Bio-Rad Radiance 2100 confocal microscope equipped with argon multi-line, green HeNe (543 nm) and red diode (637 nm) lasers. Sections were scanned through a 40× oil-immersion lens to generate z-series of 24 optical sections separated by 1 μm z-steps. In order to cover the whole of laminae I–III, it was necessary to scan six to eight z-series from each section.

The resulting confocal scans were analysed with a modification (Polgár et al., 2005, 2011) of the disector method (Williams and Rakic, 1988; Coggeshall, 1992; Bjugn and Gundersen, 1993; Guillery, 2002). NeuN and Propidium Iodide staining were initially viewed with Neurolucida for Confocal software (MicroBrightField, Colchester, VT, USA), and in each z-series the 14th optical section was designated as the reference section and the 22nd as the look-up section. Every optical section in the series was then viewed and the locations of all neuronal nuclei that were present in the reference section or appeared in subsequent sections were plotted onto an outline of the grey matter. All of those cells with nuclei that were still present in the look-up section were then excluded, leaving only those for which the bottom surface of the nucleus was located between the reference and look-up sections. The depth at which the nucleus of each of these cells was largest was also recorded, to allow subsequent comparison of the depths of nNOS^+^ and nNOS^−^ neurons in the vibratome sections (Polgár et al., 2011). nNOS-immunoreactivity was then viewed, and the presence or absence of immunostaining in each of the selected neurons was noted. Boundaries between laminae I, II and III were identified through a dark-field condenser, as described previously (Todd et al., 1998; Polgár et al., 2011) and these were plotted onto the grey matter outlines. The ventral border of lamina III was drawn from an atlas of rat spinal cord (Molander et al., 1984). In this way, we were able to determine the proportion of neurons in each lamina that were nNOS-immunoreactive. Since the number of lamina I neurons included in each of these sections was relatively low (21–39 per section), we also scanned lamina I in an additional section from each of the three animals. These were analysed in the same way, except that only neurons in lamina I were included.

Although the reference and look-up sections are relatively far apart, we ensured that all neurons with nuclei that lay between these two planes were included in the sample, by examining every optical section between them. No correction was made for tissue shrinkage, since our aim was to determine the proportion of neurons that were nNOS-immunoreactive, rather than the absolute number of cells in a volume of tissue.

### Proportion of nNOS neurons that were GABA- or PKCγ-immunoreactive

Sections from the three rats fixed with formaldehyde/glutaraldehyde were incubated in a cocktail of three primary antibodies: rabbit anti-GABA (Pow and Crook, 1993) (1:5000), guinea pig anti-PKCγ (Yoshida et al., 2006) (1:500) and sheep anti-nNOS (1:2000), which were revealed with secondary antibodies conjugated to Alexa 488, Rhodamine Red and DyLight 649, respectively.

Between five and seven vibratome sections were selected from each of the three animals before nNOS immunostaining was viewed. Either one or both dorsal horns in these sections were then scanned with the Radiance confocal microscope through a 40× objective lens to produce a set of six or seven overlapping image stacks (each consisting of 11–35 optical sections at 1 μm z-separation), such that the whole of laminae I–III was included. In this way, eight sets of scans (each corresponding to a single dorsal horn in one vibratome section) were obtained from each of the three animals. The scans were collected in such a way that the entire upper surface of laminae I–III in the vibratome section was included in these z-series.

The confocal image stacks were analysed with Neurolucida for Confocal. Since penetration of GABA staining was very limited (see below), only nNOS-immunoreactive neurons for which part of the nucleus appeared at the upper surface of the vibratome section were included. All such cells were initially selected and plotted onto an outline of the dorsal horn (as described above). The files corresponding to GABA and PKCγ were then examined and the presence or absence of each type of immunoreactivity was recorded for each of the selected nNOS cells. In order to determine whether the sizes of GABA^+^ and GABA^−^ cells differed, for each nNOS cell analysed we estimated the distance between the top of the vibratome section and the bottom of the nucleus, by counting the number of intervening confocal optical sections. Since the nucleus could be cut through any part of its z-axis, the mean of this distance should approach half of the mean z-axis length of the nucleus in a large sample of cells.

### Proportion of GABAergic boutons that were nNOS-immunoreactive

Antibody against the vesicular GABA transporter (VGAT, also known at the vesicular inhibitory amino acid transporter) was used to identify GABAergic axonal boutons (McIntire et al., 1997; Chaudhry et al., 1998; Polgár and Todd, 2008). Although VGAT is also expressed by glycinergic boutons (Chaudhry et al., 1998), most of those in laminae I–III are likely to use GABA as a co-transmitter, since virtually all glycine-immunoreactive neurons in this region are also GABA-immunoreactive (Todd and Sullivan, 1990; Polgár et al., 2003). In addition, we have shown that 96–97% of VGAT boutons in lamina II are associated with punctate (synaptic) labelling for the β3 subunit of the GABA_A_ receptor (Polgár and Todd, 2008).

Sections from three of the rats fixed with formaldehyde were incubated in sheep anti-nNOS (1:2000), rabbit anti-VGAT (Synaptic Systems, Göttingen, Germany, catalogue number 131 002; 1:1000) and guinea pig antibody against the vesicular glutamate transporter 2 (VGLUT2; Millipore, catalogue number AB5907, 1:5000), and then in secondary antibodies: anti-sheep IgG conjugated to Alexa 488, anti-guinea pig IgG conjugated to DyLight 649 and Fab′ fragment of donkey anti-rabbit IgG conjugated to Rhodamine Red (Jackson, 1:100). They were then incubated for 4 h in unconjugated Fab′ fragment of donkey anti-rabbit IgG (Jackson, 1:20), followed by 2 days in rabbit antibody against PKCγ (Santa Cruz Biotechnology, Santa Cruz, CA, USA, catalogue number sc-211; 1:1000), overnight in biotinylated donkey anti-rabbit IgG (Jackson, 1:500) and then 4 h in streptavidin conjugated to Pacific Blue (Invitrogen, 1:1000).

Two sections from each animal were selected (before nNOS immunostaining was viewed) and scanned through a 63× oil-immersion lens with a Zeiss LSM710 confocal microscope equipped with argon multi-line, blue diode (405 nm), green DPSS (561 nm) and red diode (637 nm) lasers. From each section, a set of z-series was obtained in such a way as to cover a strip ∼100 μm wide through the central part of laminae I–III from one dorsal horn (Polgár et al., 2011), with each series consisting of 25 optical sections 0.3 μm apart. The scans were analysed with Neurolucida for Confocal software. The VGAT immunostaining was initially viewed and the adjacent scans were aligned such that the full thickness of laminae I, II and III were seen. Laminar boundaries were identified based on scans obtained through a dark-field condenser (as described above), and on the position PKCγ-immunoreactive plexus, which corresponds to the inner half of lamina II (Hughes et al., 2003). The boundaries between the laminae were drawn onto an overlay, and a 5×5 μm square grid was then superimposed on the image stacks. A single optical section (the 12th in the z-series) was viewed, and 100 VGAT boutons that were present in this section were selected from each of laminae I, II and III. This was done by choosing the bouton nearest the bottom right corner of successive grid squares, starting from a square at the most dorsal part of each lamina and progressing through the squares in a dorsal-to-ventral, and then a left-to-right, direction until the 100 boutons had been acquired. Once the selection process was complete, the nNOS immunostaining was viewed, and the presence or absence of nNOS in each of the selected boutons was recorded. As this sampling method will be biased towards VGAT boutons that are more extensive in the z-axis (Guillery, 2002), we estimated the z-axis lengths of all selected boutons by determining the number of optical sections on which they appeared, and multiplying this number by 0.3 μm (the z-spacing).

We also looked for co-localisation of nNOS with VGLUT2 in these confocal image stacks, although this was not quantified, as co-localisation was very seldom seen (see below).

### nNOS in VGAT axons that were presynaptic to PKCγ cells

Sagittal sections from three rats that had been fixed with formaldehyde were incubated in the following primary antibody cocktail: guinea pig anti-PKCγ (1:500), monoclonal mouse antibody against gephyrin (Synaptic Systems, catalogue number 147 011, clone 7a; 1:1000), sheep anti-nNOS (1:2000) and rabbit anti-VGAT (1:1000). Gephyrin, nNOS and VGAT were revealed with secondary antibodies conjugated to Alexa 488, Rhodamine Red and DyLight 649, respectively, while PKCγ was detected with streptavidin–Pacific Blue conjugate (as above), following application of biotinylated anti-guinea pig secondary antibody.

One or two sections from the middle part of the dorsal horn were selected from each animal and scans through 20 PKCγ-immunoreactive neurons (10 from lamina IIi and 10 from lamina III) were obtained with the Zeiss LSM710 confocal microscope, through the 63× oil-immersion lens. Each of these scans consisted of z-series of between 14 and 28 optical sections at 0.5 μm z-separation, and in all cases, the cells were selected before the nNOS immunostaining was viewed. Scans were analysed with Neurolucida for Confocal software. For each selected PKCγ cell, the soma and any dendrites visible in the scan were first drawn. Synapses that these cells received from VGAT-immunoreactive boutons were then identified by the presence of gephyrin puncta in the membrane of the cell at sites of contact from VGAT boutons (Polgár et al., 2011), and the locations of these were recorded on the drawings. nNOS-immunostaining was then viewed, and the presence or absence of nNOS in each of the VGAT profiles that were presynaptic to the PKCγ cells was recorded.

In order to provide further evidence concerning the reliability of VGAT as a marker for inhibitory boutons, scans from two of the animals were analysed to determine the proportion of gephyrin puncta that were associated with a VGAT-immunoreactive profile. From each animal, a set of three confocal z-series, each consisting of 19 optical sections at 0.3 μm separation, was scanned through the 63× oil-immersion lens. These z-series covered a strip of tissue that included the whole of laminae I–III. The sections were viewed with Neurolucida for Confocal, and 100 gephyrin puncta were selected from each lamina in the ninth optical section in the z-series obtained from each rat. The selection was made before the VGAT channel was viewed. The VGAT was then revealed, and the presence or absence of VGAT-immunoreactive profiles adjacent to each of the selected gephyrin puncta was recorded.

### Characterization of antibodies

The nNOS antibody labels a band of 155 kDa in Western blots of rat hypothalamus, and immunostaining is abolished by pre-incubation of the antibody with nNOS (Herbison et al., 1996). The mouse monoclonal antibody NeuN was raised against cell nuclei extracted from mouse brain and found to react with a protein specific for neurons (Mullen et al., 1992). We have reported that NeuN apparently labels all neurons but no glial cells in the rat spinal dorsal horn (Todd et al., 1998). The GABA antibody was raised against GABA conjugated to porcine thyroglobulin with glutaraldehyde, and shown to be specific for GABA, with negligible cross-reactivity against other amino acids, including glutamate, aspartate, glycine or taurine (Pow and Crook, 1993). The two PKCγ antibodies were raised against peptides corresponding to the C-terminus of mouse PKCγ. The guinea pig antibody recognises a single band of appropriate molecular weight in Western blots of brain homogenates of wild-type, but not PKCγ^−/−^ mice (Yoshida et al., 2006). The rabbit VGAT antibody is directed against amino acids 75–87 of rat VGAT conjugated to keyhole limpet haemocyanin and stains bands of the appropriate molecular weight in Western blots of rat brain extracts (Takamori et al., 2000). We have reported that immunostaining in the rat dorsal horn with this antibody is abolished by pre-incubation with the immunising peptide at 10^−6^ M (Polgár et al., 2011). The gephyrin antibody was generated against an extract of rat spinal cord synaptic membranes (Pfeiffer et al., 1984). It has been extensively characterised and shown to bind to a 93 kDa peripheral membrane protein (gephyrin) in extracts of rat brain membranes (Becker et al., 1989).

### Statistical tests

The Chi-squared test, Mann–Whitney *U* test or Kruskall-Wallis ANOVA were used, as appropriate, and *P*<0.05 was taken as significant.

## Results

### nNOS immunoreactivity in laminae I–III

**Fig. 1:**
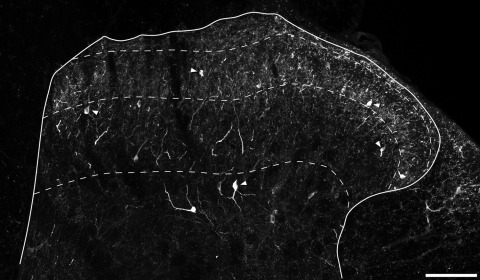
nNOS immunostaining in the dorsal horn. A confocal image from a transverse section through the L4 segment immunostained to reveal nNOS. The solid line represents the border of the grey matter, and the dashed lines show the approximate positions of the ventral borders of laminae I, II and III. There is a plexus of dendrites and axons, most of which are cut in cross section, that is most prominent in the inner part of lamina II. Scattered immunoreactive cell bodies are seen throughout the dorsal horn, and some of these are indicated with arrowheads. The image is a projection of 20 optical sections at 1 μm z-spacing. Scale bar=100 μm.

**Fig. 2:**
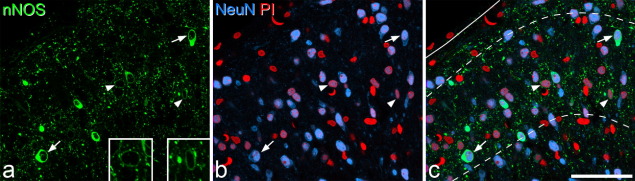
Confocal images showing nNOS-immunoreactive neurons in the superficial dorsal horn. (a) Part of a transverse section through the L4 segment immunostained for nNOS (green). (b) The same field scanned to reveal NeuN (blue) and the nuclear stain Propidium Iodide (PI, red). Neuronal nuclei appear purple, while the nuclei of non-neuronal cells (glia and capillary endothelial cells) are red. (c) The merged image, with borders between laminae I, II and III indicated with dashed lines and the dorsal edge of the grey matter shown with a solid line. Several nNOS-positive neurons are visible. Some of these show strong immunoreactivity (two marked with arrows), while others are much more weakly immunoreactive (two indicated with arrowheads). The insets in (a) show these two weakly immunoreactive cells at higher magnification. The small green profiles correspond to nNOS-containing dendrites and axons, most of which are cut in cross section. The images are from a single optical section. Scale bar=50 μm.

**Fig. 3:**
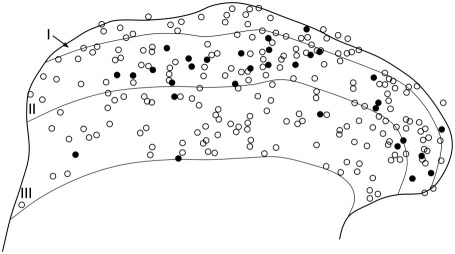
The distribution of nNOS-immunoreactive and non-immunoreactive neurons in laminae I–III. A plot of all of the neurons included in the disector sample from one of the sections that was used to determine the proportion that contained nNOS. nNOS-positive cells are shown as filled circles and nNOS-negative cells as open circles.

The distribution of immunoreactivity in the dorsal horn of the rat spinal cord was similar to the staining seen previously with nNOS antibodies, as well as to the distribution of NADPH diaphorase activity that has been reported (Valtschanoff et al., 1992a,b; Dun et al., 1993; Spike et al., 1993; Zhang et al., 1993; Herdegen et al., 1994; Laing et al., 1994; Saito et al., 1994; Wetts and Vaughn, 1994; Goff et al., 1998). nNOS-immunoreactive dendrites and axons formed a dense plexus in the superficial dorsal horn, particularly in lamina II (Fig. 1). Numerous nNOS-immunoreactive cell bodies were seen in this region, and all of these were NeuN-positive, indicating that they were neurons (Fig. 2). In all cases immunostaining was excluded from the nucleus, but occupied the perikaryal cytoplasm and often extended into proximal dendrites. The intensity of nNOS staining varied considerably between cells, with some showing intense staining and others having moderate or low levels. Results of the quantitative analysis of the proportion of neurons that were nNOS-immunoreactive are shown in Table 1, and an example of the results obtained with the disector method is illustrated in Fig. 3. nNOS was found in approximately 4% of neurons in lamina I, 18% of those in lamina II and 5% of those in lamina III.

For each of the cells included in the disector sample, we recorded the depth in the z-series at which the nucleus appeared largest. For the nNOS-immunoreactive cells, the range of depths was 6–17 μm below the top surface (median 12, *n*=148 cells), while for the non-immunoreactive cells the range was from 4 to 21 μm below this surface (median 11, *n*=1238), and these values did not differ significantly (*P*=0.21, Mann–Whitney *U* test). This suggests that there was no reduction in the proportion of neurons that were nNOS-immunoreactive at deeper levels within the z-series, which would have occurred if there was limited penetration of nNOS immunostaining into the sections.

### nNOS, GABA and PKCγ

The laminar distribution of GABA immunostaining seen in these sections was very similar to that observed previously in semithin resin-embedded sections (Todd and McKenzie, 1989; Todd and Sullivan, 1990; Spike et al., 1993; Polgár et al., 2003). However, as reported previously (Sloviter et al., 2001) its penetration into vibratome sections was extremely limited (>5 μm from the section surface). For this reason, only nNOS-positive cells for which the soma appeared at the upper surface of the vibratome section were analysed for GABA or PKCγ immunoreactivity. The distribution of PKCγ was the same as that reported previously (Mori et al., 1990; Malmberg et al., 1997; Polgár et al., 1999a; Hughes et al., 2008), with numerous immunoreactive cell bodies in the inner half of lamina II and the dorsal part of lamina III, and scattered cells elsewhere. As reported previously, very few PKCγ-immunoreactive cells were labelled with the GABA antibody (Polgár et al., 1999a).

**Fig. 4:**
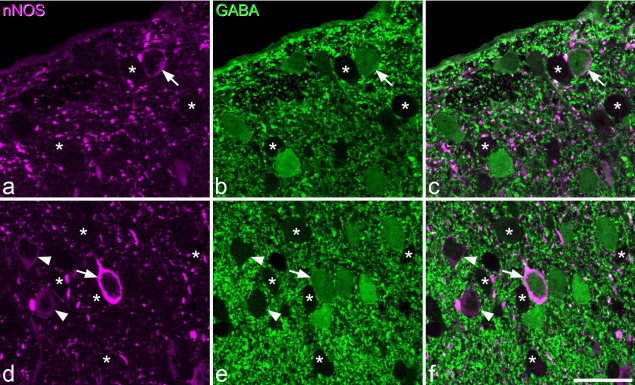
nNOS and GABA immunostaining in laminae I and II. (a–c) part of lamina I immunostained to reveal nNOS (magenta) and GABA (green). An nNOS-positive neuron that is also GABA-immunoreactive is marked with an arrow. Asterisks show examples of cells that are negative for both markers. Note that these appear as empty spaces in the GABA-stained image, due to the presence of numerous immunoreactive structures (GABA-containing axons and dendrites) in the surrounding neuropil. (d–f) part of lamina II stained in the same way. Again, an nNOS-positive cell that is GABA-immunoreactive is shown with an arrow, and some of the cells that are negative for both types of immunoreactivity are indicated with asterisks. Arrowheads show two cells with very weak nNOS staining that are GABA-negative. Note that the level of GABA in the nNOS cells is weaker than that seen in some of the nearby neurons. Images are from 4 (a–c) and 7 (d–f) optical sections at 0.3 μm z-separation. Scale bar=20 μm.

**Fig. 5:**
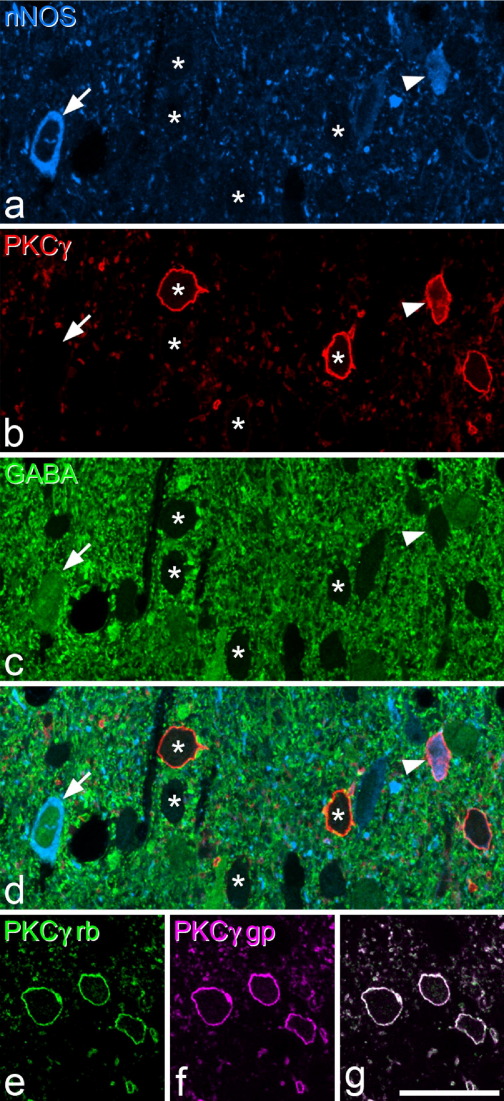
nNOS, PKCγ and GABA immunostaining. (a–c) show part of lamina III scanned to reveal nNOS (blue), PKCγ (red) and GABA (green), respectively, while (d) is a merged image of the same field. A cell that is strongly positive for nNOS, negative for PKCγ and positive for GABA is indicated with an arrow. Four cells that are negative for GABA and nNOS are marked with asterisks, and two of these are PKCγ-immunoreactive. Another PKCγ cell that is positive for nNOS and negative for GABA is indicated with an arrowhead. (e–g) comparison of immunostaining with rabbit (rb, green) and guinea pig (gp, magenta) antibodies against PKCγ in lamina II shows that both antibodies are detecting identical structures, which therefore appear white in the merged image. The images in (a–d) are from five optical sections at 0.3 μm z-separation, while those in (e–g) are from a single optical section. Scale bar=20 μm.

Quantitative data from this part of the study are shown in Table 2. All of the nNOS-positive cells in lamina I were GABA-immunoreactive (Fig. 4a–c), and none of these was PKCγ-immunoreactive. Although only 20 nNOS cells were sampled in lamina I in this part of the study, the presence of GABA-immunostaining in all of these cells indicates that at least the great majority of the nNOS cells in this lamina are GABAergic. In lamina II, 37% of the nNOS neurons sampled were GABA-immunoreactive (Fig. 4d–f), 32% were PKCγ-immunoreactive, with 1% containing both GABA- and PKCγ-immunoreactivity, and 32% containing neither. The majority of nNOS cells in lamina III were positive for GABA (50%) and/or PKCγ (53%), with 8% having both types of immunoreactivity, and only 6% having neither (Fig. 5a–d). Although many of the nNOS cells in each lamina were clearly GABA-positive, the intensity of GABA immunostaining in these cells was generally weaker than that seen in many of the surrounding neurons that lacked nNOS (Fig. 4). The mean distance between the top of the vibratome section and the bottom of the nucleus for the GABA^+^ lamina II neurons was 6.83 μm (range 1–14 μm, median 7 μm, *n*=159), while that for the GABA^−^ cells in this lamina was 5.83 μm (range 1–13 μm, median 6 μm, *n*=273), and these values differed significantly (*P*<0.005, Mann–Whitney *U* test). For lamina III neurons, the corresponding values were 5.32 μm (range 1–12 μm, median 5 μm, *n*=59; GABA^+^ cells) and 5.98 μm (range 1–12 μm, median 6 μm, *n*=61; GABA^−^ cells), and these values did not differ significantly (*P*=0.15, Mann–Whitney *U* test). The significant size difference between GABA^+^ and GABA^−^ nNOS nuclei in lamina II suggests that our sampling method was biased towards the GABA^+^ cells, as these were on average 17% longer in the z-dimension. Since the extent of the bias is directly related to this difference in z-axis length, we estimate that the true proportion of lamina II nNOS neurons that are GABAergic is 33.2%, assuming that the “corrected” number of GABA-positive cells that would have been sampled would be 136 (i.e. 159/1.17), with the same number of GABA-negative cells (273) being included.

In order to compare the nNOS immunostaining intensity between the cells that were GABA-immunoreactive and those that were not, all of the selected nNOS cells that were sampled in these three animals were assigned a score of 4 (very strong), 3 (medium), 2 (weak) or 1 (very weak) staining for nNOS. To avoid bias, this was carried out before GABA immunoreactivity was viewed. Of the 572 nNOS-positive neurons analysed, 44% were classified as very weak, 22% as weak, 15% as moderate and 20% as strong (Table 3). The proportions of neurons in each of these groups that were GABA-immunoreactive were 24%, 22%, 56% and 90%, respectively, and these were significantly different (*P*>0.001, Chi-squared test). We also determined the proportions of cells in each group that were PKCγ-immunoreactive, and found that these were 33%, 57%, 39% and 9%, respectively (Table 3). Again, these were significantly different (*P*>0.001, Chi-squared test).

To compare immunostaining with the two PKCγ antibodies, we had incubated sections from a rat fixed with formaldehyde in the rabbit and guinea pig anti-PKCγ, which were revealed with Alexa 488 and Rhodamine Red, respectively. Examination of these sections revealed that identical structures were labelled with each antibody (Fig. 5e–g).

### nNOS, VGAT and VGLUT2

**Fig. 6:**
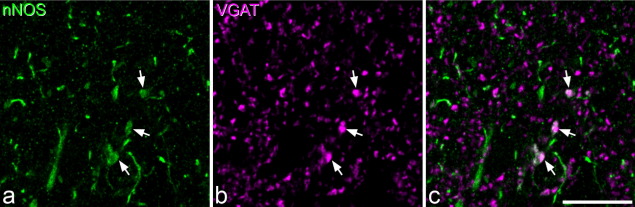
nNOS and VGAT in lamina II. Confocal images from lamina II in a transverse section reacted to reveal (a) nNOS (green) and (b) VGAT (magenta), together with a merged image (c). Three boutons that are immunoreactive with both antibodies are indicated with arrows. These are surrounded by many VGAT boutons that lack nNOS, and by scattered nNOS profiles that are not VGAT-immunoreactive. The latter include dendrites and intervaricose portions of axons. The images are projections of four optical sections at 0.3 μm z-spacing. Scale bar=10 μm.

Examination of sections that had been reacted for nNOS, VGAT, VGLUT2 and PKCγ showed that nNOS immunostaining was present in some of the VGAT-immunoreactive boutons in each lamina (Fig. 6). Quantitative analysis of 100 boutons per lamina in six sections (two from each of three rats) revealed that mean percentage of VGAT boutons that were nNOS-immunoreactive was 13.2% (range 7–18%) for lamina I, 8.2% (range 4–10%) for lamina II and 7% (range 3–10%) for lamina III. The mean of the z-axis lengths for the nNOS-positive VGAT boutons was 1.45 μm (range 0.6–3.6 μm, median 1.5 μm, *n*=170), while that for the nNOS-negative boutons was 1.41 μm (range 0.3–3.6 μm, median 1.5 μm, *n*=1630), and these did not differ significantly (*P*=0.1, Mann–Whitney *U* test). This indicates that a size difference between the two populations is unlikely to have distorted our estimate of the proportions that contained nNOS.

Although weak nNOS staining was very occasionally seen in VGLUT2-immunoreactive boutons, this was not analysed quantitatively as preliminary observation revealed that these constituted less than 1% of the VGLUT2 boutons in each lamina.

### nNOS in VGAT boutons that were presynaptic to PKCγ cells

**Fig. 7:**
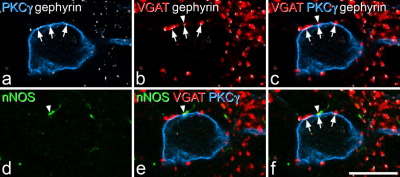
nNOS in a VGAT bouton that is presynaptic to a PKCγ neuron in a sagittal section. Confocal images from a single optical section scanned to reveal nNOS (green), VGAT (red), PKCγ (blue) and gephyrin (white). (a) The optical section passes through the cell body of a PKCγ-immunoreactive neuron in lamina III. Three gephyrin puncta in the membrane of this cell are indicated with arrows. Several other gephyrin puncta that are not associated with the cell are also visible. (b, c) Each of the gephyrin puncta in the membrane of the PKCγ cell is associated with a VGAT-positive bouton, which is presumably forming an axosomatic synapse (at the site of the gephyrin punctum). Note that virtually all of the other gephyrin puncta that are seen in this field are also in contact with VGAT boutons. (d, e) the VGAT bouton adjacent to the gephyrin punctum that is marked by the middle arrow is nNOS-immunoreactive, and its position is shown with an arrowhead in (b–f). (f) a merged image showing all four channels. Scale bar=10 μm.

Altogether, 830 VGAT boutons that contacted gephyrin puncta on the 30 PKCγ lamina II neurons (10–45 contacts per cell) and 840 VGAT boutons that contacted gephyrin puncta on the 30 PKCγ lamina III neurons (17–47 contacts per cell) were analysed. The percentage of these that were nNOS-immunoreactive varied from 0 to 14.3% (mean 2.2%, median 0%, *n*=30) for the lamina II neurons and 0–11.8% (mean 4.2%, median 3.9%, *n*=30) for the lamina III neurons. Kruskall–Wallis one-way ANOVA revealed that there was a significant difference in the percentage of VGAT boutons that were nNOS-immunoreactive between those that contacted the PKCγ cells and those in the general population in laminae II and III (*P*<0.001). Post hoc pairwise comparisons revealed a significant difference between the VGAT boutons on the PKCγ cells and those in the general population within lamina II (*P*<0.005, Mann–Whitney *U* test) but not in lamina III (*P*=0.1). An example of one of the nNOS^+^/VGAT^+^ boutons that contacted a PKCγ cell at a gephyrin punctum is shown in Fig. 7.

### Association between gephyrin puncta and VGAT boutons

Examination of the sections stained for VGAT, gephyrin, nNOS and PKCγ showed that the great majority of gephyrin puncta were associated with VGAT boutons (Fig. 7a, b). Quantitative analysis revealed that 96.5% of the selected gephyrin puncta in laminae I–III were in contact with a VGAT profile (range 95.3–97.7, *n*=300 puncta from each of two rats). Since gephyrin is associated with both GABAergic and glycinergic synapses (Fritschy et al., 2008), this finding strongly suggests that VGAT, which is known to transport both GABA and glycine (Chaudhry et al., 1998), is a reliable marker for the axonal boutons that use either or both of these transmitters in laminae I–III of the dorsal horn.

## Discussion

The main findings of this study are: (1) that nNOS is present in cell bodies of 4–5% of neurons in laminae I and III, and 18% of those in lamina II; (2) that two-thirds of nNOS-containing neurons in lamina II and half of those in lamina III are not GABA-immunoreactive, with many expressing PKCγ; and (3) that nNOS can be detected in 7–13% of VGAT^+^ boutons in these laminae but only in 2–4% of those that are presynaptic to PKCγ-immunoreactive neurons in laminae II and III.

### Comparison with previous findings

In a previous study we combined post-embedding immunocytochemistry for GABA and glycine with NADPH diaphorase histochemistry, and found that the great majority (112/123, 91%) of diaphorase-positive neurons in laminae I–III were GABA-immunoreactive, with many also showing glycine immunoreactivity (data from Fig. 1 of Spike et al., 1993). However, in the present study, only around a third of the nNOS^+^ neurons in lamina II and half of those in lamina III were GABA-immunoreactive. Immunofluorescent detection of GABA simultaneously with other antigens may be compromised by the need to avoid fixation with high concentrations of glutaraldehyde (Sloviter et al., 2001), and alternative approaches, such as *in situ* hybridisation for the 67 kDa molecular weight isoform of glutamic acid decarboxylase (GAD67) and viewing tissue from GAD67-green fluorescent protein (GFP) knock-in mice, have therefore been employed to reveal GABAergic cells (Huang et al., 2008, 2010). However, the pattern of GABA immunostaining in the present study was very similar to what we saw previously (Spike et al., 1993), and it is unlikely that the discrepancy between the two studies results from loss of sensitivity of the GABA immunostaining. A more likely explanation is that in our previous study we failed to identify cells that contained a low concentration of nNOS, due to insensitivity of the diaphorase reaction. Consistent with this, 90% of cells classified as strongly immunoreactive for nNOS in the present study were GABA-positive. It is therefore likely that only cells with high levels of nNOS were identified as NADPH diaphorase-positive in our previous study.

Because all of the neurons in laminae II–III that are not GABAergic and/or glycinergic are thought to be glutamatergic (Todd, 2010; Yasaka et al., 2010), our results suggest that many excitatory cells in this region express nNOS. One easily recognised group of excitatory interneurons consists of cells that express PKCγ (Mori et al., 1990; Malmberg et al., 1997; Polgár et al., 1999a). Hughes et al. (2008) identified a few neurons in this region that were immunoreactive for both nNOS and PKCγ, but concluded that co-localisation was a relatively rare event. In contrast, we found that ∼30% of nNOS cells in lamina II and ∼50% of those in lamina III were PKCγ-immunoreactive. Since we used the same nNOS antibody as Hughes et al., and the rabbit PKCγ antibody that they used stains identical structures to the guinea pig PKCγ antibody, it is likely that the finding of frequent co-localisation in the present study was due to the greater sensitivity of confocal microscopy. Hughes et al. may have missed weak immunostaining with one of the antibodies, particularly in cells that were strongly fluorescent with the other one. In fact, most (78%) of the PKCγ-immunoreactive neurons that were double-labelled in our study were classified as weakly or very weakly labelled for nNOS.

Ruscheweyh et al. (2006) estimated that 1–3% of lamina I neurons were nNOS-immunoreactive, based on uncorrected profile counts. The somewhat higher value that we found (4%) probably results from our use of confocal microscopy and a stereological method.

### Expression of nNOS in GABAergic and non-GABAergic neurons

We have previously reported that in the L4–5 segments of Sprague–Dawley rats 24.8%, 31.3% and 40.2% of the neurons in laminae I, II and III are GABA-immunoreactive (Polgár et al., 2003). If we assume similar values in Wistar rats, then we can estimate the proportion of GABAergic neurons in each lamina that express nNOS, based on the percentage of neurons that were nNOS-immunoreactive and the proportion of these that were GABA-immunoreactive (Table 4). According to this estimate, nNOS is likely to be present in ∼17%, 19% and 6% of the GABAergic neurons in laminae I, II and III, respectively. We have shown that nNOS, NPY, galanin and parvalbumin are present in non-overlapping neuronal populations in laminae I and II (Laing et al., 1994; Tiong et al., 2011), and have recently estimated the proportion of GABAergic neurons in each lamina that contain NPY or galanin (Polgár et al., 2011; Tiong et al., 2011). Comparison of these estimates indicates that neurons that contain nNOS, NPY or galanin constitute ∼67% of the inhibitory interneurons in lamina I and 46% of those in lamina II (Table 4). Cells that contain these three compounds are much less common in lamina III, and we found that most galanin cells in lamina III are also nNOS-immunoreactive (Tiong et al., 2011). Parvalbumin-containing cells are present in laminae II and III, but the proportion of GABAergic neurons in these laminae that contain parvalbumin is not known.

Glutamatergic cells make up around ∼69% of the neuronal population in lamina II (Polgár et al., 2003). Based on our finding that nNOS was present in 18% of lamina II neurons and that 67% of these were not GABA-immunoreactive, we estimate that 17% of the excitatory interneurons in this lamina express nNOS. A similar calculation suggests that ∼4% of glutamatergic neurons in lamina III contain nNOS. In lamina III nNOS was found mainly in GABA- or PKCγ-immunoreactive neurons, with only 6% of the cells being negative for both markers. However, in lamina II, around 32% of the nNOS neurons were neither GABA- nor PKCγ-immunoreactive. This implies that nNOS is expressed in several neuronal populations: GABAergic inhibitory interneurons throughout laminae I–III, and at least two types of excitatory interneuron in laminae II–III: those with and those without PKCγ.

### nNOS-immunoreactive axons

Although our results indicate that many glutamatergic cells in laminae II and III express nNOS, nNOS-immunoreactivity was very seldom seen in boutons that contained VGLUT2, which is expressed by glutamatergic neurons in this region (Oliveira et al., 2003; Todd et al., 2003; Alvarez et al., 2004; Llewellyn-Smith et al., 2007; Yasaka et al., 2010). This is consistent with the results obtained with electron microscopy by Valtschanoff et al. (1992b), who reported that all nNOS-containing axon terminals in lamina II are GABA-immunoreactive. The most likely explanation is that nNOS is only present at detectable levels in axons of cells that have a high concentration in the soma, most of which are GABAergic. Since the majority (90/112) of GABA-immunoreactive NADPH diaphorase-positive neurons in laminae I–III were also glycine-immunoreactive (Spike et al., 1993), it is likely that many of the nNOS^+^/VGAT^+^ boutons use GABA and glycine as co-transmitters. However, for convenience they will be referred to as GABAergic.

Immunocytochemical studies have provided evidence for innervation of different types of dorsal horn neuron by specific neurochemical types of GABAergic axon. For example, we reported that 27% of gephyrin puncta (presumed inhibitory synapses) on giant lamina I spinoparabrachial neurons were associated with nNOS-immunoreactive boutons, while for lamina I neurons with the NK1 receptor (NK1r), which are also likely to have been projection cells (Al Ghamdi et al., 2009), nNOS^+^ boutons were present at only 3% of their gephyrin puncta (Puskár et al., 2001). Since the present study shows that 13% of VGAT boutons in lamina I are nNOS-immunoreactive, this indicates that nNOS-containing interneurons preferentially innervate the giant cells, but are substantially under-represented among inhibitory inputs to NK1r-expressing neurons in this lamina. NK1r-expressing projection neurons in lamina III also receive very few contacts from nNOS-immunoreactive boutons, but are targeted by axons that contain NPY and GABA, which provide >30% of their inhibitory synapses (Polgár et al., 1999b, 2011). We recently reported that excitatory interneurons with PKCγ in lamina IIi receive ∼36% of their inhibitory input from NPY/GABA axons (Polgár et al., 2011). The inputs from NPY-containing axons to both the lamina III NK1r projection neurons and the PKCγ^+^ interneurons represent a selective innervation, since only 15% of VGAT^+^ boutons in lamina II, and 5% of those in lamina III, were NPY-immunoreactive. The present results indicate that although nNOS-containing GABAergic boutons occasionally form synapses with the PKCγ cells, they are significantly under-represented among the inhibitory inputs to those in lamina II.

### Functions of nNOS in the superficial dorsal horn

NO generated by nNOS acts on soluble guanylate cyclase (sGC) to produce cyclic guanosine monophosphate (cGMP) (Garthwaite, 2008). Although NO and cGMP in the spinal cord have no effect on acute pain thresholds, studies with NOS inhibitors and knockout mice indicate that NO derived from nNOS is required for development and maintenance of hyperalgesia in inflammatory and neuropathic pain states (Malmberg and Yaksh, 1993; Meller and Gebhart, 1993; Osborne and Coderre, 1999; Luo and Cizkova, 2000; Tao et al., 2004; Chu et al., 2005; Boettger et al., 2007; Guan et al., 2007). There is also evidence that NO can have anti-nociceptive effects within the spinal cord (Zhuo et al., 1993; Iwamoto and Marion, 1994), and it has been suggested that the concentration determines whether it is pro- or antinociceptive (Schmidtko et al., 2009).

NO acts both pre- and postsynaptically (Garthwaite, 2008). Presynaptic actions involve release from nNOS-containing axons, leading to depolarisation or hyperpolarisation of the postsynaptic target (Garthwaite, 2008). A postsynaptic mechanism can result from activation of nNOS in cell bodies or dendrites by Ca^2+^ entering through NMDA receptors, and there is thought to be a direct interaction between nNOS and the NMDA receptor NR2B subunit (Brenman et al., 1996), which is present at many glutamatergic synapses in the superficial dorsal horn (Nagy et al., 2004). It has been proposed that in this situation NO can act as a retrograde signal, leading to prolonged changes in synaptic strength (Steinert et al., 2010), and there is evidence that it is required for development of long-term potentiation in the superficial dorsal horn (Zhang et al., 2005; Ikeda et al., 2006).

Since NO diffuses from its site of production and is able to cross membranes, its effects are dependent on the distance that it can travel from its site of synthesis and the location of sGC. Ding and Weinberg (2006) have reported that the β1 subunit of sGC is present throughout the dorsal horn, and is expressed by NK1 receptor-expressing projection neurons in lamina I, as well as by both excitatory and inhibitory interneurons in the superficial laminae. Interestingly, although nNOS- and sGC-immunoreactive structures were frequently found in contact with each other, in most cases both structures lacked synaptophysin (a marker for axonal boutons), suggesting NO-mediated dendro-dendritic interactions. However, some nNOS-containing axons were associated with sGC-containing profiles, and this arrangement presumably underlies the presynaptic actions of NO. Taken together with the findings of Valtschanoff et al. (1992b) the present results indicate that the axons in this situation are nearly all GABAergic, and these are presumably derived from local inhibitory interneurons that show strong nNOS immunoreactivity. In addition, Ding and Weinberg (2006) found sGC in axonal boutons that were in contact with nNOS profiles. If some of these boutons are glutamatergic, this arrangement could underlie the post-synaptic actions of NO described above. Our finding that nNOS was present in GABA-immunoreactive and GABA-negative cells indicates that this mechanism could operate at synapses on both inhibitory and excitatory interneurons.

Several studies have found up-regulation of nNOS in neurons in the dorsal horn during the development of inflammatory pain states, and it has been reported that there is an increase in the number of immunoreactive cells, suggesting *de novo* expression (Herdegen et al., 1994; Yonehara et al., 1997; Maihöfner et al., 2000; Chu et al., 2005). However, since we saw many neurons with very weak nNOS immunoreactivity, it is possible that the apparent increase in numbers of immunoreactive cells reported in these studies is due to an up-regulation of nNOS in interneurons that normally express it at very low levels.

## Conclusion

Laminae I–III of the dorsal horn contain a large number of inhibitory and excitatory interneurons, and in order to understand their roles in neuronal circuits we need to identify functional populations within each of these broad groups. Although morphological and electrophysiological approaches have defined certain distinct types, for example GABAergic islet cells and glutmatergic vertical and radial cells in lamina II (Grudt and Perl, 2002; Maxwell et al., 2007; Graham et al., 2007; Yasaka et al., 2010), many of the inhibitory and excitatory interneurons have defied classification. This region is also highly complex in terms of its neurochemistry, since the expression of neuropeptides, certain receptors, calcium-binding proteins and several enzymes is restricted to specific populations of neurons (Todd, 2010). Despite this complexity, the use of appropriate combinations of neurochemical markers has allowed us to define discrete classes of inhibitory and excitatory interneurons, and some of these have been shown to be selective in their postsynaptic targets (Polgár et al., 1999b, 2011; Puskár et al., 2001). It will be important in future studies to integrate the results obtained from morphological and physiological studies with these neurochemical findings, for example by carrying out electrophysiological recordings from mice in which expression of GFP is linked to different neurochemical markers that define populations of interneurons in the superficial dorsal horn.

## Figures and Tables

**Table 1:** Percentages of neurons in laminae I–III that were nNOS-immunoreactive

Lamina	Number of neurons counted	Number of nNOS^+^ cells	% of neurons that were nNOS^+^
I	80.3 (68–91)	3.3 (2–4)	4.2 (3.2–5.1)
II	216.3 (201–232)	38 (32–42)	17.6 (13.8–20.5)
III	165.3 (141–182)	8 (6–10)	4.8 (3.5–5.6)

In each case the mean values for the three animals are shown, with the range in parentheses.

**Table 2:** Percentages of nNOS neurons sampled in laminae I–III that were GABA- and/or PKCγ-immunoreactive

Lamina	Number of nNOS^+^ cells counted	% that were GABA^+^	% that were PKCγ^+^	% that were GABA^+^ and PKCγ^+^	% that were GABA^−^ and PKCγ^−^
I	6.7 (3–9)	100 (100–100)	0 (0–0)	0 (0–0)	0 (0–0)
II	144 (121–164)	36.7 (34.7–40.1)	32.3 (24.4–47.9)	1 (0–1.7)	32 (19–40.2)
III	40 (30–47)	49.6 (46.5–53.3)	52.5 (44.7–62.8)	8.2 (6.4–11.6)	6.1 (2.3–12.8)

In each case the mean values for the three animals are shown, with the range in parentheses.

**Table 3:** Strength of nNOS immunostaining in GABA^+^ and PKCγ^+^ cells in laminae I–III

nNOS strength	Number	% GABA^+^	% PKCγ^+^
1	251	24%	33%
2	123	22%	57%
3	85	56%	39%
4	113	90%	9%
Total	572		

The table shows the number of nNOS neurons of each staining intensity, and the percentages of these that were immunoreactive for GABA or PKCγ (data pooled from three animals). nNOS strength was defined as very weak (1), weak (2), medium (3) or strong (4).

**Table 4:** Estimated percentages of GABAergic neurons that contain nNOS, NPY or galanin

Lamina	% of neurons that are nNOS^+^	% of nNOS neurons that are GABA^+^	% of neurons that are nNOS^+^/GABA^+^	% neurons that are GABAergic^a^	% of GABAergic neurons that contain nNOS	% of GABAergic neurons that contain NPY^b^	% of GABAergic neurons that contain galanin^c^
I	4.2	100	4.2	24.8	16.9	23.4	26.4
II	17.6	33.2^d^	5.8	31.3	18.7	17.3	9.9
III	4.8	49.6	2.4	40.2	5.9	9.5	4.9

a The percentages of neurons that are GABAergic are taken from data for the L4–5 segments of three naive Sprague–Dawley rats examined by Polgár et al. (2003).

b Data from Polgár et al. (2011).

c Data from Tiong et al. (2011).

d The estimate for the percentage of nNOS neurons in lamina II that are GABA-immunoreactive has been corrected for the size difference between GABA^+^ and GABA^−^ neurons among the nNOS-immunoreactive cells in this lamina (see text).
